# Survival and Treatment of Lung Cancer in Taiwan between 2010 and 2016

**DOI:** 10.3390/jcm10204675

**Published:** 2021-10-12

**Authors:** Yen-Jung Chang, Jing-Yang Huang, Ching-Hsiung Lin, Bing-Yen Wang

**Affiliations:** 1 Department of Otorhinolaryngology, Head and Neck Surgery, Changhua Christian Hospital, Changhua 50006, Taiwan; 182912@cch.org.tw; 2 Institute of Medicine, Chung Shan Medical University, Taichung 40201, Taiwan; wchinyang@gmail.com; 3 Center for Health Data Science, Chung Shan Medical University Hospital, Taichung 40201, Taiwan; 4 Department of Recreation and Holistic Wellness, MingDao University, Changhua 523008, Taiwan; 47822@cch.org.tw; 5 Department of Internal Medicine, Division of Chest Medicine, Changhua Christian Hospital, Changhua 50006, Taiwan; 6 Institute of Genomics and Bioinformatics, National Chung Hsing University, Taichung 40227, Taiwan; 7 Department of Surgery, Division of Thoracic Surgery, Changhua Christian Hospital, Changhua 50006, Taiwan; 8 School of Medicine, Chung Shan Medical University, Taichung 40201, Taiwan; 9 School of Medicine, College of Medicine, Kaohsiung Medical University, Kaohsiung 80708, Taiwan; 10 College of Medicine, National Chung Hsing University, Taichung 40227, Taiwan; 11 Center for General Education, MingDao University, Changhua 523008, Taiwan

**Keywords:** lung cancer, stage, treatment, surgery

## Abstract

Background: Lung cancer is the leading cause of cancer-related death, and its incidence is still growing in Taiwan. This study investigated the prognostic factors of overall survival between 2010 and 2016 in Taiwan. Methods: Data from 2010 to 2016 was collected from the Taiwan Cancer Registry (TCR). The characteristics and overall survival of 71,334 lung cancer patients were analyzed according to the tumor, node, metastasis (TNM) 7th staging system. Univariate and multivariate analysis were performed to identify the prognostic factors. Results: The five-year overall survival (*n* = 71,334) was 25.0%, and the median survival was 25.3 months. The five-year overall survival of patients receiving any kind of treatment (*n* = 65,436; 91.7%) and surgical resection (*n* = 20,131; 28.2%) was 27.09% and 69.93%, respectively. The clinical staging distribution was as follows: stage IA (9208, 12.9%), stage IB (4087, 5.7%), stage IIA (1702, 2.4%), stage IIB (1454, 2.0%), stage IIIA (5309, 7.4%), stage IIIB (6316, 8.9%), stage IV (41458, 58.1%). Age, sex, Charlson comorbidity index, cell type, clinical T, clinical N, clinical M, grading and treatment strategy are independent prognostic factors in the multivariate analysis. Conclusion: The outcome for lung cancer patients was still poor. The identification of prognostic factors could facilitate in choosing treatment strategies and designing further randomized clinical trials.

## 1. Introduction

Lung cancer is the leading cause of cancer-related deaths worldwide [1,2,3]. Most patients are diagnosed at advanced stages, and long-term survival remains poor. In the CONCORD-3 program, the age-standardized five-year net survival for patients diagnosed with lung cancer during 2010–2014 was in the 10–20% range in most countries [4]. For over 23 million people in Taiwan, lung cancer has also been the leading cause of cancer-related deaths and is still growing [2,5,6]. The incidence in men and women increased from 22.5 to 43.5 and from 9.5 to 31.6 cases per 100,000, respectively, from 1986 to 2017 [6]. Only 14% to 18% of patients with lung cancer are alive within five years after diagnosis [7]. Because of the growth of lung cancer incidence and deaths throughout the years, the identification of prognostic factors is important for patients and physicians. Recognition of prognostic factors could help physicians to choose adequate treatment strategies. Understanding the current status of lung cancer can help scholars make appropriate clinical research experiments.

We have investigated lung cancer and prognosis based on the tumor, node, metastasis (TNM) 6th edition in Taiwan from 2002 to 2008 [5]. During the study period, the five-year survival rate was only 15.9% and the median survival was 13.2 months. Approximately 84% of patients were diagnosed at stage III or stage IV. We identified age, sex, tumor location, cell type and differentiation as independent prognostic factors [5]. The 7th edition of the TNM classification for lung cancer was released in 2009 [8], and the 8th edition was updated in 2016 [9]. The Taiwan Cancer Registry (TCR) adopted the 7th TNM stages between 2010 and 2016. The prognosis and treatment outcome of lung cancer patients evolved over time. Therefore, we re-analyzed the patient characteristics, treatment outcome and trends of lung cancer in Taiwan between 2010 and 2016 according to the 7th TNM stages. We performed univariate and multivariate analysis to identify the independent prognostic factors.

## 2. Patients and Methods

The study was approved by the Internal Review Board of Changhua Christian Hospital (Internal Review Board number 161222). The TCR [10] was implemented in 1979, and the recorded number of clinical variables was extended from 20 to 114 in 2011 [10]. The TCR is maintained by the Ministry of Health and Welfare of Taiwan. Under government commission, novice training courses and support to registrars have been standardized, and field data audits have been performed periodically through medical chart review. There is an excellent level of completeness (97%) [10]. Hundreds of researchers use the database because of its completeness of principal diagnostic and demographic information. Enrolled patients in the TCR must be confirmed by pathologic malignancy. The TCR was also linked to the National Health Insurance of Taiwan. The National Health Insurance of Taiwan covered most treatment-related costs, including blood analysis, chest/abdominal computed tomography scans, positron emission tomography/computed tomography scans, operative costs, chemotherapy, target therapy and radiotherapy in Taiwan.

Records for the lung cancer patients were retrieved from the TCR by the International Classification of Diseases for Oncology (ICD-O) site codes C34.0, C34.1, C34.2, C34.3, C34.8, and C34.9. All data was collected from the TCR and was related to new diagnoses between 2010 and 2016. Follow-up data was used from the date of diagnosis to either the date of death or the censoring date of 31 December 2018. The following clinical variables were included into the study for analysis: age, sex, Charlson Comorbidity Index, cell type, clinical T, clinical N, clinical M, clinical stage, grading, treatment strategy and survival data. The initial treatment strategy was defined as the therapy administrated within three months of diagnosis. All tumor specimens were graded histologically based on the World Health Organization’s classification of lung cancers and were staged according to the 7th TNM staging system [8].

### Statistical Analysis

The analysis for this study was done using SAS System (Statistical Analysis Software 9.4, SAS Institute Inc., Cary, NC, USA) for Windows. Overall survival was defined from tissue diagnosis to the date of death or 31 December 2018. Date of death and its cause were obtained from a Taiwan death certificate database. The overall survival curve was calculated by the Kaplan-Meier method, and the log-rank test was used to determine significant differences. The Charlson Comorbidity Index [11] was used to quantify pre-existing comorbidities.

To investigate the prognostic factors of overall survival in lung cancer patients, all of the following clinical items were included in both the univariable analysis and multivariable analysis: age, sex, Charlson Comorbidity Index, cell type, clinical T, clinical N, clinical M, clinical stage, grading, and treatment strategy. Analysis was performed by using the Cox proportional hazards model. A probability value less than 0.05 was considered to be significant. All statistical calculations were performed by a biostatistician (Jing Yang Huang).

## 3. Results

**Figure 1:**
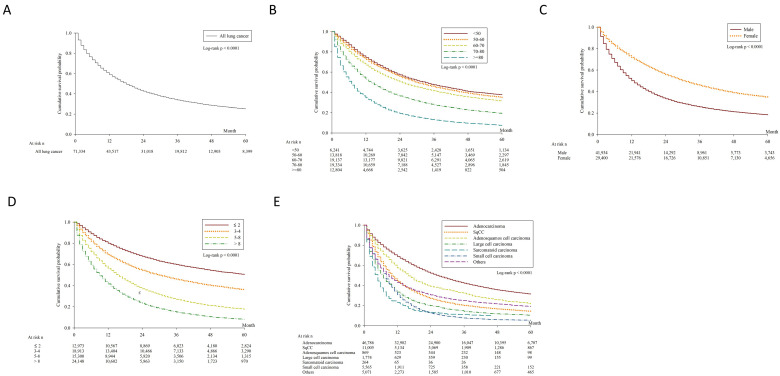
Kaplan-Meier overall survival. (**A**) Kaplan-Meier survival curves for 71,334 patients with lung cancer. (**B**) Kaplan-Meier survival curves stratified by age (*p* < 0.0001). (**C**) Kaplan-Meier survival curves stratified by sex (*p* < 0.0001). (**D**) Kaplan-Meier survival curves stratified by Charlson comorbidity index score (*p* < 0.0001). (**E**) Kaplan-Meier survival curves stratified by histologic cell type (*p* < 0.0001).

Throughout the study, a total of 71,334 lung cancer patients were included, of which 58.8% (*n* = 41,934) were men. The five-year OS was 25% and the median survival was 25.3 months (Figure 1A). The clinical demographic data, five-year survival, median survival time and univariate survival analysis are summarized in Table 1. The five-year overall survival was assessed and stratified according to each clinical variable. Univariable survival analysis indicated that age, sex, Charlson Comorbidity Index, cell type, clinical T, clinical N, clinical stage, grading, treatment strategy and surgical method were prognostic factors. The overall survival curve was plotted and stratified according to each clinical variable (age, sex, Charlson comorbidity index, cell type, clinical stage, clinical T, clinical N, clinical M, treatment strategy and surgical method).

The five-year overall survival rates according to age were 37.6% (<50 years), 35.0% (50–60 years), 31.35% (60–70 years), 19.28% (70–80 years) and 7.46% (≥80 years). As age increases, overall survival gets worse (Figure 1B). Five-year survival was lower in men (18.23%) than in women (34.71%) (Figure 1C). The survival rates stratified by the Charlson Comorbidity Index were shown in Figure 1D, and the difference was also significant. The most frequent cell type was adenocarcinoma (*n* = 46,786, 65.6%), followed by squamous cell carcinoma (*n* = 11,005, 15.4%), small cell carcinoma (*n* = 5565, 7.8%), large cell carcinoma (*n* = 1774, 2.5%), adenosquamous cell carcinoma (*n* = 869, 1.2%) and sarcomatoid carcinoma (*n* = 264, 0.4%). Adenocarcinoma had a significantly superior 5-year survival rate (31.25%) compared to other cell types (Figure 1E).

**Figure 2:**
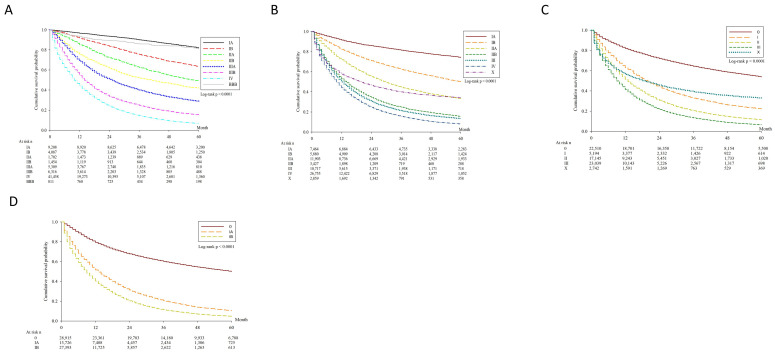
Kaplan-Meier overall survival. (**A**) Kaplan-Meier survival curves stratified by clinical stage (*p* < 0.0001). (**B**) Kaplan-Meier survival curves stratified by clinical T stage (*p* < 0.0001). (**C**) Kaplan-Meier survival curves stratified by clinical N stage (*p* < 0.0001). (**D**) Kaplan-Meier survival curves stratified by clinical M stage (*p* < 0.0001).

The clinical staging was distributed as follows: stage IA (9208, 12.9%), stage IB (4087, 5.7%), stage IIA (1702, 2.4%), stage IIB (1454, 2.0%), stage IIIA (5309, 7.4%), stage IIIB (6316, 8.9%), stage IV (41,458, 58.1%). Significant differences in survival were noted between neighboring stages (Figure 2A). The survival curve was also assessed based on the clinical T (Figure 2B), clinical N (Figure 2C) and clinical M (Figure 2D) stages, respectively. 

**Figure 3:**
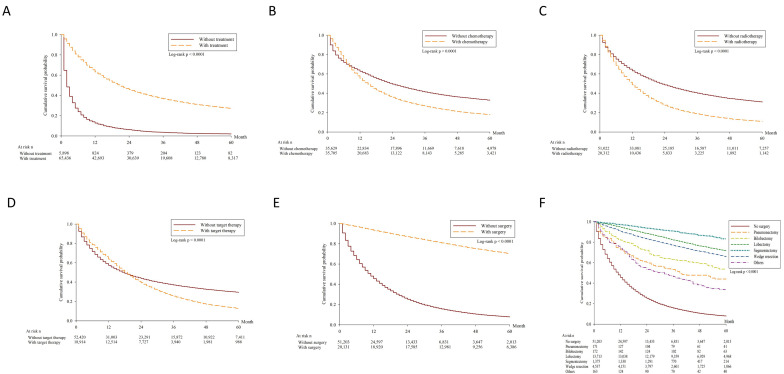
Kaplan-Meier overall survival. (**A**) Kaplan-Meier survival curves according to with or without any treatment (*p* < 0.0001). (**B**) Kaplan-Meier survival curves according to with or without chemotherapy (*p* < 0.0001). (**C**) Kaplan-Meier survival curves according to with or without radiotherapy (*p* < 0.0001). (**D**) Kaplan-Meier survival curves according to with or without target therapy (*p* < 0.0001). (**E**) Kaplan-Meier survival curves according to with or without surgery (*p* < 0.0001). (**F**) Kaplan-Meier survival curves stratified by surgical method (*p* < 0.0001).

The five-year OS of patients receiving any kind of treatment (*n* = 65,436; 91.7%) is 27.09%. The survival curves were plotted according to treatment (yes vs. no), chemotherapy (yes vs. no), radiotherapy (yes vs. no), target therapy (yes vs. no) and surgery (yes vs. no) (Figure 3A–E). Of the 20,131 patients undergoing pulmonary resection, 171 patients (0.85%) had pneumonectomies, 172 patients (0.85%) had bilobectomies, 13,713 patients (68.12%) had lobectomies, 1375 patients (6.83%) had segmentectomies, 4537 patients (22.54%) had wedge resections, and 163 patients (0.81%) had other surgical interventions. Patients receiving survival resection had a five-year survival rate of 69.93%, and among the various surgical methods patients who underwent segmentectomy had the best five-year survival rate (Figure 3F).

Multivariable analysis of overall survival showed age, sex, Charlson Comorbidity Index, cell type, clinical T, clinical N, clinical M, grading and treatment strategy are independent prognostic factors (Table 2).

The characteristics of patients receiving surgery and univariate survival analysis are summarized in Supplement Table S1. Multivariable analysis of overall survival were shown in Supplement Table S2. The survival curve of patients receiving surgery was plotted in Supplement Figure S1A. The survival curve was also assessed according to the pathologic stage (Supplement Figure S1B), pathologic T (Supplement Figure S1C, pathologic N (Supplement Figure S1D) and pathologic M (Supplement Figure S1E) stages, respectively. The treatment effectiveness and survival were stratified by different specific clinical stages (Supplement Tables S3–S6).

## 4. Discussion

This study describes the characteristics of lung cancer in Taiwan using the 7th TNM system between 2010 and 2016. The five-year OS was 25.0%, and the median survival was 25.3 months. Age, sex, CCI, cell type, clinical T, clinical N, clinical M, grading, chemotherapy, target therapy, and surgical resection were identified as independent prognostic factors.

The National Lung Screening Trial reported that screening with the use of low-dose CT reduces lung cancer-specific mortality by 20.0% in high-risk smokers [12,13].There was still no lung cancer screening program covered by Taiwan health insurance. Recently, Taiwan’s health promotion administration announced the proposal of lung cancer screening in 2021. They recommended adults aged 50 to 80 years who have a 30 pack-year smoking history or family history. The time for this policy to be implemented is still undetermined.

With the increasing use of computer tomography, more early-stage lung cancers are found. In Taiwan between 2010 and 2016, 18.6% of lung cancer cases were clinical stage I. In our previous study between 2002 and 2008, only 12.5% of the patients had clinical stage I lung cancer [5]. Patients with early stage, potentially curable lung cancer could have timely treatments and thereby improve their overall prognosis. Therefore, the five-year survival rate of lung cancer increased from 15.9% in the 2002 to 2008 study to 25% in this 2010 to 2016 study. The stage at diagnosis is an important prognostic factor. The early detection of lung cancer is the most effective way to improve the survival rate of lung cancer patients.

Regarding stage IV lung cancer, there have only been small improvements in five-year survival rates among patients even though there have been advances in systemic drug therapy (4.91% between 2002 and 2008; 6.65% between 2010 and 2016). Most patients were diagnosed at stage IV (*n* = 41,458, 58.1%) and the five-year overall survival rate is only 6.65%. Therapeutic progress for advanced lung cancer is driven by molecularly targeted therapeutics, immune checkpoint inhibitors, and anti-angiogenic agents [14,15,16]. Molecular testing is recommended to be conducted in cases of advanced lung cancer. Molecular and immunologic data could help physicians to determine treatment strategies. All of the new systemic treatments significantly contributed to a better prognosis of lung cancer over time.

During the study period (2010–2016), a total of 20,131 patients (28.2%) received surgical resection, with a five-year survival rate of 69.93%. In our previous study (2002–2008), only 16.4% of patients underwent surgery and the five-year survival rate was 57.19%. Complete surgical resection provided the highest curative probability for lung cancer. As the proportion of operations increases, the survival rate of lung cancer can also be increased in Taiwan. Some studies have reported resection rates between 8.8% and 19.7% [17,18,19]. Survival varied among different population databases due to various cancer stages and treatment modalities. For patients with lung cancer, increasing the rate of surgery can help improve survival.

Lobectomy remains the standard surgical method for treatment of early-stage disease [20], although some selected patients with poor pulmonary function or other major comorbidities that contraindicate lobectomy are treated with sublobar resection. Among the surgical group (*n* = 20,131) in the study, lobectomy (*n* = 13,713, 68.1%) and wedge resection (*n* = 4537, 22.5%) were performed most often in Taiwan between 2010 and 2016. The proportion of lobectomy rose from 60.5% (2002–2008) [5] to 68.1% (2010–2016). Sublobar resection, either segmentectomy or by wide wedge resection, is appropriately performed in patients with peripherally located cT1N0M0 lung cancers smaller than 2 cm in diameter [21,22]. In our current study, patients who underwent segmentectomy (*n* = 1375, 6.85%) had the best five-year survival rate compared with patients who underwent other procedures. Segmentectomy is a valuable and promising surgical method. Two prospective, randomized, and multi-institutional phase III trials are being conducted to establish the effectiveness of intentional sublobar resections (segmentectomy/wedge resection) for small peripheral lung cancer [23,24,25]. The patient performance, clinical stage, tumor size and the consolidation/tumor ratio were the crucial factors in the selection of the surgical method. In Taiwan, segmentectomy is appropriately performed in patients with peripherally located cT1N0M0 lung cancers or patients who could not tolerate lobectomy due to limited performance status. In the study, patients who underwent segmentectomy had the best five-year survival rate compared with patients who underwent other procedures, because these patients receiving segmentectomy in Taiwan were highly selected. The study design method could not conclude that segmentectomy provided the superior survival outcome compared to other surgical methods. Our results only demonstrate the current treatment outcome. It is far too early to consider segmentectomy as the preferable surgical method in lung cancer according to current evidence.

In our study, older age at diagnosis was an unfavorable factor in multivariate analysis. With increasing age, overall survival gets worse. The impact of age on lung survival has been controversial [26,27,28]. The five-year overall survival rates in Taiwan according to age were 37.6% (<50 years), 35.0% (50–60 years), 31.35% (60–70 years), 19.28% (70–80 years) and 7.46% (≥80 years). Patients older than 70 years have significant survival decline compared with those younger than 70. Physicians should understand the influence of age on survival. There may be limited performance status and poorer tolerance to treatment in older patients.

The influence of sex on survival is still inclusive [29,30]. Sex is an independent prognostic factor in our analysis. The male/female incidence was 1.43 and the five-year survival was 18.23% in men compared to 34.71% in women between 2010 and 2016. In our previous study between 2002 and 2008 [5], the male/female lung cancer incidence ratio was 1.90 and the five-year overall survival rates were 13.28% and 20.67% in males and females, respectively. The incidence in men and women increased from 22.5 to 43.5 and from 9.5 to 31.6 cases per 100,000, respectively, in Taiwan from 1986 to 2017 [6]. Lung cancer is increasing at a faster rate in women than in men, and the improvement of women’s treatment performance was significantly higher than that of men in Taiwan. The lung cancer incidences for males and females have also converged in America and several other countries, especially among the younger generation [31,32]. The differential incidence and survival based on sex might be attributed to sex-specific differences in the pathogenesis of lung cancer.

The influence of lung cancer cell type on survival was still undetermined [33,34]. Most studies investigated the difference between squamous cell carcinoma and adenocarcinoma, for they are the most known subtypes of lung cancer. We have analyzed 37,463 patients with adenocarcinoma or squamous cell carcinoma and performed propensity matching to adjust unbalanced clinical data [35]. We considered that adenocarcinoma and squamous cell carcinoma have significantly different characteristics and outcomes. In this current study, subtypes were put into more specific groups. With a bigger database, the results remain the same, where adenocarcinoma (five-year survival of 31.25%) had a better outcome than any other subtype (14.33% in squamous cell carcinoma, 21.58% in adenosquamous cell carcinoma, 10.12% in large cell carcinoma, and 9.16% in sarcomatoid carcinoma). The pathogenesis and survival among different lung cancer cell types were also different, as were the treatment protocols and clinical guidelines for lung cancer [21]. In esophageal cancer, for example, adenocarcinoma and squamous cell carcinoma have distinctly different risk factors, pathogeneses, and treatment modalities and they have been separated since the 7th edition of the TNM staging system [36]. We may assume that different lung cancer cell types have different pathogeneses and clinical disease courses. Further clinical studies should be designed based on the same histology.

The International Association for the Study of Lung Cancer (IASLC)/American Thoracic Society (ATS)/European Respiratory Society (ERS) international multidisciplinary classification of lung adenocarcinoma was published in 2011 [37]. Multiple studies have demonstrated that the adenocarcinoma classification has predictive value [38,39,40]. Lepidic-predominant adenocarcinoma has almost as favorable a prognosis, acinar/papillary-predominant adenocarcinomas are intermediate risk, and micropapillary/solid-predominant adenocarcinomas have the worst survival rates. A growing proportion of lepidic-predominant adenocarcinoma at the time of diagnosis might be surmised to have a better survival rate over time. However, the subtypes of adenocarcinoma were not available in the Taiwan Cancer Registry, which is one if the Registry’s limitations.

In the univariable analysis, if patients without chemotherapy or target therapy have favorable survival rates, it means patients receiving systemic treatment had advanced lung cancer. On the other hand, multivariate analysis showed a decreased risk of death in patients treated with chemotherapy or target therapy. That reflected the treatment effectiveness of chemotherapy or target therapy after adjusting clinical stages. Different treatment options were selected according to clinical staging, and clinical stage could affect survival directly. Therefore, the prognostic factors such as treatment methods should be interpreted cautiously and carefully.

One strength of the study is its large number of patients, which contributed to its ample power to identify minor differences between subgroups. The study included all lung cancer cases in Taiwan. There were several limitations in the study, including its retrospective nature, older TNM stages, few variables, and its inability to capture neoadjuvant treatment and potential confounders. The potential confounders, such as health literacy, education level, performance status, tobacco smoking status, immunotherapy, adenocarcinoma subtypes and mutation status were not recorded in the database. Confounders may influence the results, and the results should be interpreted with caution. The chemotherapy regimens, target therapy, surgical skills and radiotherapy protocols varied among different hospitals in Taiwan. The heterogeneous treatment protocols may also have confounded the study results.

In conclusion, the survival of lung cancer patients remains unsatisfactorily low. Early detection and increasing the rate of surgical resection may improve survival rates. The identification of prognostic factors could facilitate the choosing of treatment strategies and the designing of further randomized clinical trials.

## Figures and Tables

**Table 1:** Patient clinical data and univariate survival analysis.

Variable	Number of Patients	5-Year Survival(%, 95% C.I.)	Median Survival Time(Months, 95% C.I.)	*p*
All	71,334	25.00 (24.64–25.35)	25.27 (24.62–25.92)	
Age (years)				<0.0001
<50	6241	37.59 (36.26–38.92)	33.28 (31.72–34.85)	
50–60	13,818	35.07 (34.18–35.96)	30.45 (29.52–31.38)	
60–70	19,137	31.35 (30.60–32.09)	25.27 (24.62–25.92)	
70–80	19,334	19.28 (18.66–19.90)	14.51 (14.08–14.94)	
≥80	12,804	7.46 (6.96–7.99)	7.26 (7.03–7.48)	
Sex				<0.0001
Male	41,934	18.23 (17.82–18.64)	13.02 (12.79–13.26)	
Female	29,400	34.71 (34.10–35.33)	31.08 (30.36–31.80)	
CCI				<0.0001
≤2	12,973	50.35 (49.38–51.32)	61.62 (58.57–64.66)	
3–4	18,913	36.04 (35.28–36.80)	30.61 (29.56–31.66)	
5–8	15,300	17.55 (16.87–18.23)	16.15 (15.70–16.60)	
>8	24,148	8.01 (7.63–8.41)	9.74 (9.54–9.94)	
Cell type				<0.0001
Adenocarcinoma	46,786	31.25 (30.77–31.72)	27.08 (26.60–27.55)	
SqCC	11,005	14.33 (13.62–15.04)	10.77 (10.47–11.07)	
Adenosquamous cell carcinoma	869	21.58 (18.59–24.72)	16.75 (15.14–18.36)	
Large cell carcinoma	1774	10.12 (8.67–11.70)	7.28 (6.67–7.89)	
Sarcomatoid carcinoma	264	9.16 (6.02–13.09)	5.12 (4.18–6.05)	
Small cell carcinoma	5565	5.31 (4.69–5.98)	8.35 (8.11–8.59)	
Others or unknown	5071	19.04 (17.87–20.24)	9.43 (8.90–9.97)	
Clinical T				<0.0001
1a	7464	74.11 (72.95–75.23)	Not estimated	
1b	5880	49.72 (48.27–51.16)	60.19 (57.94–62.44)	
2a	11,903	33.10 (32.16–34.05)	29.91 (29.00–30.82)	
2b	3427	15.37 (14.05–16.75)	14.34 (13.29–15.38)	
3	10,717	13.40 (12.69–14.14)	12.93 (12.56–13.29)	
4	26,755	7.93 (7.56–8.32)	10.69 (10.48–10.90)	
X	2859	33.80 (31.85–35.77)	20.31 (18.13–22.50)	
Missing	2329	24.48 (22.64–26.35)	16.98 (14.83–19.13)	
Clinical N				<0.0001
0	22,510	53.77 (53.03–54.50)	72.44 (70.55–74.34)	
1	5194	22.18 (20.93–23.46)	20.29 (19.27–21.31)	
2	17,145	11.48 (10.94–12.03)	13.61 (13.29–13.92)	
3	23,039	6.56 (6.19–6.94)	9.93 (9.73–10.12)	
X	2742	32.71 (30.75–34.69)	19.10 (16.53–21.67)	
Missing	704	41.43 (37.63–45.18)	31.01 (22.91–39.11)	
Clinical M				<0.0001
0	28,915	49.92 (49.28–50.56)	60.46 (59.21–61.72)	
1A	13,726	10.38 (9.80–10.99)	13.73 (13.34–14.11)	
1B	27,393	4.68 (4.39–4.99)	9.50 (9.32–9.68)	
B	816	80.67 (77.04–83.79)	Not estimated	
Missing	484	28.91 (24.74–33.21)	14.33 (11.94–16.73)	
Clinical stage				
IA	9208	81.77 (80.80–82.70)	Not estimated	
IB	4087	63.13 (61.41–64.79)	94.34 (88.00–100.67)	
IIA	1702	48.78 (46.10–51.41)	57.74 (46.73–68.75)	
IIB	1454	41.55 (38.75–44.32)	41.17 (38.29–44.05)	
IIIA	5309	28.59 (27.26–29.94)	25.49 (24.40–26.59)	
IIIB	6316	15.32 (14.33–16.34)	14.83 (14.30–15.36)	
IV	41,458	6.65 (6.37–6.94)	10.70 (10.53–10.87)	
BBB	811	80.96 (77.33–84.08)	Not estimated	
Unknown	989	61.10 (57.77–64.26)	Not estimated	
Grade				<0.0001
Well differentiated	5056	71.24 (69.83–72.60)	Not estimated	
Moderately differentiated	17,163	45.63 (44.78–46.47)	49.77 (47.73–51.82)	
Poorly differentiated	13,520	20.27 (19.50–21.06)	14.74 (14.27–15.21)	
Undifferentiated	434	23.27 (19.17–27.62)	12.30 (10.26–14.34)	
Missing	35,161	10.39 (10.04–10.76)	11.98 (11.78–12.19)	
Treatment				<0.0001
Any treatment	65,436	27.09 (26.71–27.47)	21.28 (20.97–21.58)	
Chemotherapy	35,705	17.77 (17.34–18.21)	15.37 (15.12–15.62)	
Surgery	20,131	69.93 (69.19–70.66)	Not estimated	
Radiotherapy	20,312	10.79 (10.32–11.28)	12.51 (12.26–12.77)	
Target therapy	18,914	12.62 (12.05–13.21)	19.10 (18.74–19.46)	
Surgical method				<0.0001
No surgery	51,203	7.70 (7.43–7.97)	11.29 (11.13–11.44)	
Pneumonectomy	171	43.99 (35.99–51.69)	39.55 (35.98–43.11)	
Bilobectomy	172	53.67 (45.56–61.09)	74.77 (68.17–81.38)	
Lobectomy	13,713	71.16 (70.29–72.01)	Not estimated	
Segmentectomy	1375	83.37 (80.29–86.01)	Not estimated	
Wedge resection	4537	65.72 (64.02–67.36)	103.30 (101.87–104.73)	
Others	163	33.58 (25.83–41.48)	32.18 (26.26–38.10)	

CCI = Charlson comorbidity index; CI = confidence interval; M = metastasis; N = node; T = tumor; SqCC = squamous cell carcinoma.

**Table 2:** Multivariate analysis of overall survival.

Variable	aHR	95% Confidence Interval	*p*-Value
Age			
<50	0.901	0.851–0.955	0.0005
50–60	0.906	0.867–0.946	<0.0001
60–70 (reference)	1		
70–80	1.387	1.336–1.439	<0.0001
≥80	1.960	1.877–2.046	<0.0001
Sex			
Male	1.459	1.412–1.506	<0.0001
Female (reference)	1		
Charlson score			
≤2	0.859	0.820–0.900	<0.0001
3–4 (reference)	1		
5–8	1.233	1.185–1.283	<0.0001
>8	1.388	1.337–1.441	<0.0001
Cell type			
Adenocarcinoma (reference)	1		
SqCC	1.392	1.337–1.448	<0.0001
Adenosquamous cell carcinoma	1.552	1.397–1.724	<0.0001
Large cell carcinoma	1.632	1.496–1.781	<0.0001
Sarcomatoid carcinoma	2.394	1.937–2.960	<0.0001
Small cell carcinoma	1.296	1.224–1.373	<0.0001
Others	1.261	1.176–1.352	<0.0001
Clinical T			
1A	1		
1B	1.552	1.430–1.684	<0.0001
2A	1.889	1.755–2.033	<0.0001
2B	2.331	2.134–2.546	<0.0001
3	2.278	2.108–2.461	<0.0001
4	2.357	2.185–2.541	<0.0001
X	2.149	1.844–2.504	<0.0001
Clinical N			
0	1		
1	1.312	1.239–1.389	<0.0001
2	1.503	1.438–1.571	<0.0001
3	1.677	1.603–1.754	<0.0001
X	1.645	1.435–1.885	<0.0001
Clinical M			
0	1		
1A	1.503	1.434–1.576	<0.0001
1B	2.091	2.006–2.178	<0.0001
B	0.351	0.278–0.444	<0.0001
Differentiation			
Well (reference)	1		
Moderate	1.365	1.287–1.447	<0.0001
Poor	1.632	1.536–1.733	<0.0001
Undifferentiated	1.772	1.559–2.015	<0.0001
Surgery			
No (reference)	1		
Yes	0.370	0.352–0.389	<0.0001
Chemotherapy			
No (reference)	1		
Yes	0.812	0.784–0.842	<0.0001
Radiotherapy			
No (reference)	1		
Yes	1.021	0.989–1.055	0.1983
Target therapy			
No (reference)	1		
Yes	0.651	0.623–0.681	<0.0001

aHR = adjusted hazard ratio.

## Data Availability

Restrictions apply to the availability of these data. Data was obtained from Taiwan Cancer Registry and are available from Bing-Yen Wang with the permission of Taiwan Cancer Registry.

## Supplementary Materials

The following are available online at https://www.mdpi.com/article/10.3390/jcm10204675/s1, Figure S1: (A) Kaplan-Meier survival curves for 20131 lung cancer patients receiving surgery. (B) Kaplan-Meier survival curves stratified by pathologic stage (P < 0.0001). (C) Kaplan-Meier survival curves stratified by pathologic T stage (*P* < 0.0001). (D) Kaplan-Meier survival curves stratified by pathologic N stage (P < 0.0001). (E) Kaplan-Meier survival curves stratified by pathologic M stage (P < 0.0001), Table S1: The characteristics of patient receiving surgery and univariate survival analysis, Table S2: Multivariate analysis of overall survival of patient receiving surgery, Table S3: Treatment modalities and survival in clinical stage I patients, Table S4: Treatment modalities and survival in clinical stage II patients, Table S5: Treatment modalities and survival in clinical stage III patients, Table S6: Treatment modalities and survival in clinical stage IV patients.
