# Supplementary material for: Survival and Treatment of Lung Cancer in Taiwan between 2010 and 2016
Source: J Clin Med. 2021 Oct 12;10(20):4675. doi: 10.3390/jcm10204675 (PMC8540538; doi:10.3390/jcm10204675)
Supplement: Supplementary file 1 [file jcm-10-04675-s001.zip › jcm-1393855-supplementary/Supplement data.pdf]

**Supplement Table S1**. The characteristics of patient receiving surgery and univariate survival analysis

| Variable | Number of Patients | 5-Year Survival (%, 95% C.I.) | Median Survival Time (months, 95% C.I.) | *p* |
|---|---|---|---|---|
| All | 20131 | 69.93 (69.19-70.66) | Not estimated | |
| Age (years) | | | | <.0001 |
| <50 | 2397 | 80.64 (78.77-82.37) | Not estimated | |
| 50-60 | 5145 | 77.03 (75.66-78.33) | Not estimated | |
| 60-70 | 6569 | 72.72 (71.43-73.97) | Not estimated | |
| 70-80 | 4741 | 60.01 (58.38-61.59) | 83.01 (78.26-87.75) | |
| ≧80 | 1279 | 45.51 (42.34-48.62) | 56.06 (53.31-58.81) | |
| Sex | | | | <.0001 |
| Male | 9801 | 59.86 (58.74-60.96) | 90.68 (87.91-93.45) | |
| Female | 10330 | 79.68 (78.75-80.58) | Not estimated | |
| CCI | | | | <.0001 |
| ≦2 | 7016 | 81.87 (80.77-82.92) | Not estimated | |
| 3-4 | 7893 | 71.98 (70.82-73.11) | Not estimated | |
| 5-8 | 3149 | 57.17 (55.15-59.14) | 76.42 (71.04-81.80) | |
| >8 | 2073 | 43.47 (41.05-45.87) | 47.18 (44.48-49.88) | |
| Cell type | | | | <.0001 |
| Adenocarcinoma | 15828 | 75.07 (74.26-75.86) | Not estimated | |
| SqCC | 2368 | 48.12 (45.89-50.32) | 57.13 (53.68-60.59) | |
| Adenosquamous cell carcinoma | 382 | 42.79 (37.08-48.38) | 47.70 (43.52-51.87) | |
| Large cell carcinoma | 314 | 37.01 (31.31-42.70) | 28.12 (25.36-30.88) | |
| Sarcomatoid carcinoma | 50 | 43.20 (29.15-56.48) | 30.94 (24.41-37.47) | |
| Small cell carcinoma | 206 | 36.30 (29.43-43.19) | 24.50 (20.98-28.02) | |
| Others or unknown | 983 | 72.05 (68.64-75.16) | Not estimated | |
| Grade | | | | <.0001 |
| Well differentiated | 3890 | 88.16 (86.93-89.29) | Not estimated | |
| Moderately differentiated | 9989 | 71.11 (70.04-72.14) | Not estimated | |
| Poorly differentiated | 4033 | 53.68 (51.85-55.47) | 67.51 (65.61-69.40) | |
| Undifferentiated | 194 | 46.79 (39.08-54.12) | 52.53 (48.14-56.92) | |
| Missing | 2025 | 63.01 (60.60-65.32) | Not estimated | |
| Clinical stage | | | | <.0001 |
| IA | 8771 | 84.35 (83.41-85.25) | Not estimated | |
| IB | 3508 | 70.69 (68.90-72.41) | Not estimated | |

| | | | | |
|---|---|---|---|---|
| IIA | 1292 | 57.91 (54.78-60.90) | 79.61 (74.66-84.57) | |
| IIB | 944 | 57.64 (54.04-61.06) | 85.58 (78.98-92.18) | |
| IIIA | 2280 | 48.95 (46.61-51.25) | 58.61 (55.86-61.35) | |
| IIIB | 624 | 43.91 (39.40-48.32) | 48.66 (43.56-53.76) | |
| IV | 1258 | 33.76 (30.86-36.68) | 34.99 (31.20-38.78) | |
| BBB | 759 | 85.57 (82.00-88.48) | Not estimated | |
| Unknown | 695 | 75.87 (72.14-79.17) | Not estimated | |
| Pathologic | | | | <.0001 |
| IA | 8244 | 87.68 (86.78-88.52) | Not estimated | |
| IB | 4616 | 74.98 (73.49-76.40) | Not estimated | |
| IIA | 1454 | 57.80 (54.83-60.65) | 77.40 (65.78-89.02) | |
| IIB | 956 | 54.81 (51.27-58.20) | 74.18 (69.36-79.00) | |
| IIIA | 2463 | 45.33 (43.07-47.55) | 53.55 (51.29-55.82) | |
| IIIB | 150 | 41.53 (32.95-49.87) | 42.59 (39.46-45.72) | |
| IV | 1452 | 33.92 (31.17-36.68) | 36.35 (34.13-38.57) | |
| BBB | 527 | 56.13 (51.07-60.89) | 80.84 (76.77-84.91) | |
| Unknown | 269 | 60.14 (53.43-66.19) | 90.33 (86.55-94.11) | |
| Adjuvant Treatment | | | | |
| Chemotherapy | 5452 | 65.28 (63.79-66.72) | Not estimated | |
| Radiotherapy | 1126 | 45.04 (41.71-48.31) | 53.01 (50.52-55.50) | |
| Target therapy | 238 | 61.26 (53.62-68.02) | Not estimated | |
| Surgical method | | | | |
| Pneumonectomy | 171 | 43.99 (35.99-51.69) | 39.55 (35.98-43.11) | |
| Bilobectomy | 172 | 53.67 (45.56-61.09) | 74.77 (68.17-81.38) | |
| Lobectomy | 13,713 | 71.16 (70.29-72.01) | Not estimated | |
| Segmentectomy | 1375 | 83.37 (80.29-86.01) | Not estimated | |
| Wedge resection | 4537 | 65.72 (64.02-67.36) | 103.30 (101.87-104.73) | |
| Others | 163 | 33.58 (25.83-41.48) | 32.18 (26.26-38.10) | |

CCI= Charlson comorbidity index; CI = confidence interval; M = metastasis; N = node; T = tumor; SqCC=squamous cell carcinoma

**Supplement Table S2**. Multivariate analysis of overall survival of patient receiving surgery

| Variable | aHR | 95% Confidence Interval | *p* value |
|---|---|---|---|
| Age | | | |
| <50 | 0.693 | 0.622-0.771 | <.0001 |
| 50-60 | 0.846 | 0.784-0.914 | <.0001 |
| 60-70 (reference) | 1 | | |
| 70-80 | 1.496 | 1.398-1.601 | <.0001 |
| ≧80 | 2.021 | 1.841-2.219 | <.0001 |
| Sex | | | |
| Male | 1.564 | 1.474-1.659 | <.0001 |
| Female (reference) | | | |
| Charlson score | | | |
| ≦2 | 0.824 | 0.766-0.886 | <.0001 |
| 3-4 (reference) | 1 | | |
| 5-8 | 1.290 | 1.202-1.385 | <.0001 |
| >8 | 1.398 | 1.295-1.510 | <.0001 |
| Cell type | | | |
| Adenocarcinoma (reference) | 1 | | |
| SqCC | 1.447 | 1.346-1.555 | <.0001 |
| Adenosquamous cell carcinoma | 1.867 | 1.622-2.149 | <.0001 |
| Large cell carcinoma | 1.930 | 1.654-2.252 | <.0001 |
| Sarcomatoid carcinoma | 1.798 | 1.237-2.615 | 0.0021 |
| Small cell carcinoma | 2.268 | 1.896-2.712 | <.0001 |
| Others | 0.965 | 0.845-1.102 | 0.5979 |
| Pathologic stage | | | |
| IA (reference) | 1 | | |
| IB | 1.809 | 1.650-1.984 | <.0001 |
| IIA | 2.980 | 2.651-3.349 | <.0001 |
| IIB | 3.585 | 3.165-4.062 | <.0001 |
| IIIA | 4.803 | 4.338-5.319 | <.0001 |
| IIIB | 5.289 | 4.228-6.617 | <.0001 |
| IV | 6.115 | 5.545-6.743 | <.0001 |
| Differentiation | | | |
| Well (reference) | 1 | | |
| Moderate | 1.566 | 1.415-1.735 | <.0001 |
| Poor | 1.966 | 1.761-2.195 | <.0001 |

| | | | |
|---|---|---|---|
| Undifferentiated | 1.758 | 1.395-2.216 | <.0001 |
| Adjuvant chemotherapy | | | |
| No (reference) | 1 | | |
| Yes | 0.772 | 0.722-0.825 | <.0001 |
| Adjuvant radiotherapy | | | |
| No (reference) | 1 | | |
| Yes | 1.049 | 0.953-1.154 | 0.3302 |
| Adjuvant target therapy | | | |
| No (reference) | 1 | | |
| Yes | 0.881 | 0.705-1.101 | 0.2641 |
| Surgical method | | | |
| Pneumonectomy | 1.391 | 1.133-1.707 | 0.0016 |
| Bilobectomy | 1.117 | 0.895-1.394 | 0.3265 |
| Lobectomy (reference) | 1 | | |
| Segmentectomy | 0.797 | 0.681-0.933 | 0.0047 |
| Wedge resection | 1.386 | 1.294-1.485 | <.0001 |
| Others | 1.956 | 1.583-2.417 | <.0001 |

aHR = adjusted hazard ratio

**Supplement Table S3**. Treatment modalities and survival in clinical stage I patients

| Variable | Number of Patients | 5-Year Survival (%, 95% C.I.) | Median Survival Time (months, 95% C.I.) | *p for* 5-Year Survival |
|---|---|---|---|---|
| Treatment | | | | |
| Any treatment | | | | <.0001 |
| No | 173 | 15.44 (10.36-21.44) | 13.83 (9.54-18.13) | |
| Yes | 13122 | 76.76 (75.89-77.60) | Not estimated | |
| Chemotherapy | | | | <.0001 |
| No | 9827 | 78.95 (77.98-79.88) | Not estimated | |
| Yes | 3468 | 67.64 (65.80-69.41) | Not estimated | |
| Surgery | | | | <.0001 |
| No | 1016 | 24.96 (22.07-27.95) | 28.04 (26.47-29.61) | |
| Yes | 12279 | 80.34 (79.48-81.16) | Not estimated | |
| Radiotherapy | | | | <.0001 |
| No | 12434 | 78.54 (77.67-79.38) | Not estimated | |
| Yes | 861 | 39.14 (35.44-42.81) | 41.37 (38.93-43.82) | |
| Target therapy | | | | <.0001 |
| No | 12934 | 76.58 (75.71-77.43) | Not estimated | |
| Yes | 361 | 52.77 (46.66-58.50) | 63.96 (56.32-71.60) | |

CI = confidence interval

**Supplement Table S4**. Treatment modalities and survival in clinical stage II patients

| Variable | Number of Patients | 5-Year Survival (%, 95% C.I.) | Median Survival Time (months, 95% C.I.) | *p for* 5-Year Survival |
|---|---|---|---|---|
| Treatment | | | | |
| Any treatment | | | | <.0001 |
| No | 148 | 8.60 (4.68-14.00) | 8.50 (7.01-9.99) | |
| Yes | 3008 | 47.29 (45.29-49.26) | 54.19 (51.60-56.78) | |
| Chemotherapy | | | | 0.0008 |
| No | 1287 | 41.86 (38.89-44.80) | 40.82 (36.21-45.42) | |
| Yes | 1869 | 47.92 (45.37-50.43) | 54.86 (52.13-57.58) | |
| Surgery | | | | <.0001 |
| No | 920 | 15.63 (13.15-18.31) | 15.81 (14.85-16.77) | |
| Yes | 2236 | 57.79 (55.44-60.06) | 81.37 (77.34-85.40) | |
| Radiotherapy | | | | <.0001 |
| No | 2406 | 50.53 (48.27-52.73) | 61.38 (59.21-63.55) | |
| Yes | 750 | 29.23 (25.72-32.83) | 25.63 (21.28-29.98) | |
| Target therapy | | | | <.0001 |
| No | 2976 | 46.12 (44.13-48.09) | 52.06 (49.70-54.41) | |
| Yes | 180 | 33.12 (24.94-41.51) | 37.59 (27.82-47.37) | |

CI = confidence interval

**Supplement Table S5**. Treatment modalities and survival in clinical stage III patients

| Variable | Number of Patients | 5-Year Survival (%, 95% C.I.) | Median Survival Time (months, 95% C.I.) | *p for* 5-Year Survival |
|---|---|---|---|---|
| Treatment | | | | |
| Any treatment | | | | <.0001 |
| No | 782 | 2.62 (1.63-3.98) | 4.84 (4.28-5.40) | |
| Yes | 10843 | 22.79 (21.91-23.67) | 20.09 (19.51-20.66) | |
| Chemotherapy | | | | 0.7884 |
| No | 3349 | 21.58 (20.03-23.16) | 15.78 (14.50-17.07) | |
| Yes | 8276 | 21.36 (20.39-22.35) | 19.33 (18.64-20.02) | |
| Surgery | | | | <.0001 |
| No | 8721 | 12.68 (11.91-13.47) | 13.92 (13.50-14.33) | |
| Yes | 2904 | 47.88 (45.80-49.92) | 57.01 (54.46-59.57) | |
| Radiotherapy | | | | <.0001 |
| No | 6394 | 23.73 (22.59-24.90) | 19.26 (18.34-20.17) | |
| Yes | 5231 | 18.61 (17.45-19.80) | 17.68 (17.07-18.29) | |
| Target therapy | | | | 0.0005 |
| No | 10078 | 20.86 (19.99-21.74) | 17.23 (16.71-17.74) | |
| Yes | 1547 | 24.77 (22.30-27.32) | 29.31 (26.95-31.68) | |

CI = confidence interval

**Supplement Table S6**. Treatment modalities and survival in clinical stage IV patients

| Variable | Number of Patients | 5-Year Survival (%, 95% C.I.) | Median Survival Time (months, 95% C.I.) | *p for* 5-Year Survival |
|---|---|---|---|---|
| Treatment | | | | |
| Any treatment | | | | <.0001 |
| No | 4660 | 0.84 (0.59-1.16) | 2.60 (2.52-2.68) | |
| Yes | 36798 | 7.39 (7.08-7.71) | 12.45 (12.26-12.65) | |
| Chemotherapy | | | | <.0001 |
| No | 19802 | 7.87 (7.40-8.34) | 10.50 (10.14-10.86) | |
| Yes | 21656 | 5.58 (5.25-5.93) | 10.80 (10.62-10.98) | |
| Surgery | | | | <.0001 |
| No | 40200 | 5.77 (5.50-6.05) | 10.38 (10.21-10.55) | |
| Yes | 1258 | 33.76 (30.86-36.68) | 34.99 (31.20-38.78) | |
| Radiotherapy | | | | <.0001 |
| No | 28101 | 7.63 (7.27-8.00) | 11.31 (11.08-11.54) | |
| Yes | 13357 | 4.60 (4.19-5.03) | 9.77 (9.55-9.99) | |
| Target therapy | | | | <.0001 |
| No | 24727 | 4.27 (4.00-4.55) | 7.45 (7.30-7.59) | |
| Yes | 16731 | 10.10 (9.54-10.68) | 18.03 (17.68-18.39) | |

CI = confidence interval

**Supplement Figure legends**

Figure S1. (A) Kaplan-Meier survival curves for 20131 lung cancer patients receiving surgery. (B) Kaplan-Meier survival curves stratified by pathologic stage (P < 0.0001). (C) Kaplan-Meier survival curves stratified by pathologic T stage ($P < 0.0001$). (D) Kaplan-Meier survival curves stratified by pathologic N stage (P < 0.0001). (E) Kaplan-Meier survival curves stratified by pathologic M stage (P < 0.0001).
