# Supplementary figures and images for: Survival and Treatment of Lung Cancer in Taiwan between 2010 and 2016
Source: J Clin Med. 2021 Oct 12;10(20):4675. doi: 10.3390/jcm10204675 (PMC8540538; doi:10.3390/jcm10204675)

## Slide 1
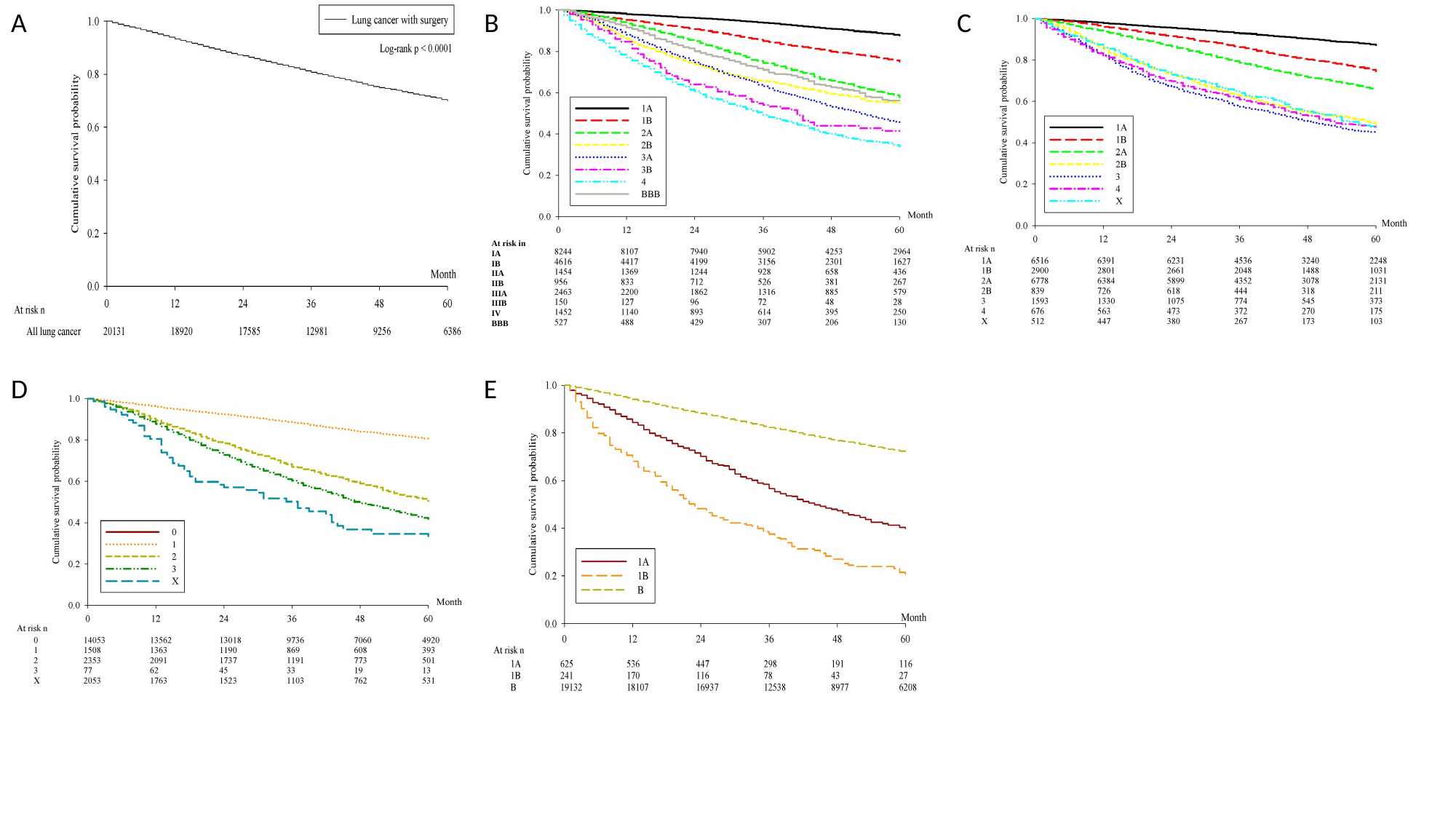

A
B
C
At risk in
IA
IB
IIA
IIB
IIIA
IIIB
IV
BBB
D
E

Supplement: Supplementary file 1 [file jcm-10-04675-s001.zip › jcm-1393855-supplementary/Supplement figure S1.pptx]
